# HOTTIP Predicts Poor Survival in Gastric Cancer Patients and Contributes to Cisplatin Resistance by Sponging miR-216a-5p

**DOI:** 10.3389/fcell.2020.00348

**Published:** 2020-05-08

**Authors:** Rui Zhao, Xin Zhang, Yanli Zhang, Yaping Zhang, Yongmei Yang, Yue Sun, Xin Zheng, Ailin Qu, Yvette Umwali, Yi Zhang

**Affiliations:** ^1^Department of Clinical Laboratory, Qilu Hospital of Shandong University, Jinan, China; ^2^Key Laboratory of Tumor Marker Translational Medicine, Shandong Provincial Medicine and Health, Jinan, China; ^3^Department of Clinical Laboratory, Shandong Provincial Third Hospital, Jinan, China

**Keywords:** gastric cancer, HOTTIP, miR-216a-5p, autophagy, chemoresistance

## Abstract

Gastric cancer (GC) is a significant public health burden worldwide, and cisplatin resistance is the leading cause for the failure of chemotherapy in this disease. Previous studies have revealed that HOXA transcript at the distal tip (HOTTIP) is involved in the pathology of GC and is associated with poor overall survival. However, the functional role of HOTTIP in GC chemoresistance remains unclear. In this study, quantitative real-time PCR was used to analyze HOTTIP expression in GC cell lines and in tissues of GC patients who received cisplatin-based chemotherapy. The mechanism of HOTTIP-mediated chemoresistance was assessed using cell viability, apoptosis, and autophagy assays. The relationships among HOTTIP, miR-216a-5p, and Bcl-2 were determined using luciferase reporter and western blot assays. HOTTIP was markedly upregulated in the tissues of GC patients who were treated with gastrectomy and cisplatin chemotherapy, especially in those with recurrent tumors. Further, HOTTIP was increased in the cisplatin-resistant cell line, SGC7901/DDP, compared to the parental cells, SGC7901. Functional assays demonstrated that HOTTIP expression promoted cisplatin resistance and inhibited apoptosis and autophagy in GC cells. Mechanistic investigations revealed that HOTTIP may regulate the functions of GC cells by sponging miR-216a-5p. MiR-216a-5p overexpression decreased Bcl-2 expression, enhanced Beclin1 expression, and active autophagy. Taken together, our study demonstrated that HOTTIP is closely associated with recurrence in GC patients. HOTTIP expression confers cisplatin resistance by regulating the miR-216a-5p/BCL-2/Beclin1/autophagy pathway, which provides a novel strategy to overcome resistance to chemotherapy in GC.

## Introduction

Gastric cancer (GC) is a leading cause of cancer-related deaths worldwide, accounting for over 720,000 deaths annually (Torre et al., 2015). Despite several advances in the treatment methods, patients with GC have extremely poor outcome marked by a 5-year survival rate of less than 30% (Allemani et al., 2018). Platinum-based chemotherapy has been widely used to inhibit tumor growth and metastasis, and improve the prognosis and quality of life of patients with GC (Ajani et al., 2016; Wagner et al., 2017). However, GC patients often develop drug resistance, which greatly affects the therapeutic effect of the drug and may eventually cause tumor recurrence (Hosoda et al., 2019). Therefore, it is important to study the chemoresistance mechanism in GC, which may help to improve the efficacy of chemotherapy in GC.

The Encyclopedia of DNA Elements (ENCODE) project has dramatically altered our understanding of the role of non-coding RNAs in human physiology and pathology (Harrow et al., 2012; Pennisi, 2012). HOTTIP (HOXA transcript at the distal tip) is a tumor-related long non-coding RNA (lncRNA) located at the chromosomal locus 7p15.2 (Wang et al., 2011). Our previous study showed that HOTTIP was markedly upregulated in the serum of GC patients and positively associated with tumor invasion depth, advanced TNM stage, and poor overall survival (Zhao et al., 2018). Ye et al. (2016) also found increased HOTTIP expression in GC tissues and cell lines, and correlated it with aggressive clinicopathological characteristics of GC patients. Chang et al. (2016) reported that HOTTIP was an oncogene in GC progression and downregulation of HOTTIP inhibits cell growth and impairs cell invasion and migration by reducing the expression of HOXA13. Recently, Sun et al. (2018) found that HOTTIP participates in tumor chemoresistance by acting as a competing endogenous RNA (ceRNA). However, the biological function and molecular mechanism of HOTTIP in chemoresistance in GC remain unclear.

Recent studies have revealed that autophagy plays an important role in regulating response to chemotherapy (Janku et al., 2011). Autophagy is a self-proteolytic cellular degradation process in which damaged proteins and organelles are delivered to lysosomes for degradation (Mehrpour et al., 2010). This process eliminates potentially dangerous material, maintains cellular homeostasis, and may help cancer cells survive. However, excessive autophagy may promote cell death (Galluzzi et al., 2012). Xu et al. (2019) showed that inhibition of autophagy significantly decreased Dex-induced tumor cell death and promoted chemoresistance in multiple myeloma. Evidence suggests that HOTTIP overexpression can inhibit autophagy in renal cell carcinoma (RCC) cells through the PI3K/Akt/Atg13 signaling pathway (Su et al., 2019). However, whether HOTTIP can modulate chemoresistance in GC by regulating autophagy remains largely unknown.

In this study, we identified that HOTTIP was significantly associated with recurrence in GC patients who received cisplatin-based chemotherapy. Through gain or loss of function studies, we demonstrated that HOTTIP enhanced cisplatin resistance and inhibited autophagy and apoptosis in GC cells. Mechanistically, HOTTIP acted as an endogenous “sponge” for miR-216a-5p and inhibited autophagy by regulating the Bcl-2/Beclin1 signaling pathway. Therefore, targeting HOTTIP represents a potential therapeutic strategy to reduce chemoresistance in GC.

## Materials and Methods

### Human Tissues

The study cohort comprised 106 patients with stage II/III GC who underwent gastrectomy with standard D2 lymph node resection at the Qilu Hospital of Shandong University (Jinan, China) between January 2008 and December 2012. Clinical staging of GC was performed based on the 8th edition TNM staging system and the detail clinical information was shown in Supplementary Table S1. The assessment of response was performed according to Response Evaluation Criteria in Solid Tumors (RECIST) criteria. The main eligibility criteria included (1) age 18–75 years, (2) histologically proven gastric cancer, (3) no prior chemotherapy or radiation, and (4) no other serious disease (except GC). Exclusion criteria included (1) concomitant cancers, (2) significant comorbid conditions, (3) could not comply with the protocol, without signed informed consent or withdrawal of consent, (4) incomplete medical records or lost to follow-up, and (5) women who were pregnant or breast feeding. Adjacent normal tissues were used as healthy controls, and these adjacent tissues were confirmed to be devoid of tumor cells by pathologists. The tissue samples were immediately frozen and stored at −150°C until analysis. All patients provided written informed consent and the Ethics Committee of the Qilu Hospital of Shandong University approved this study.

All patients were followed up at 3-month intervals for the first 2 year, at 6-month intervals for the next 3 years, and the date of retrieval of the latest record was December 31, 2017. Disease-free survival (DFS) was defined as the interval between receiving surgery and recurrence. Overall survival (OS) was defined as the period from the time of surgery to death.

### Cell Culture

The human normal gastric epithelial cell line (GES-1) and GC cell line (SGC7901) were purchased from the BeNa Culture Collection (Shanghai, China). Cells were cultured in RPMI-1640 medium (Hyclone, Logan, UT, United States) supplemented with 10% fetal bovine serum (Sigma-Aldrich, St. Louis, MO, United States) and 1% penicillin-streptomycin (Solarbio, Beijing, China) at 37°C in a humidified atmosphere containing 5% CO_2_.

The cisplatin-resistant adenocarcinoma cell line from human stomach, SGC7901/DDP, was established by exposing parental SGC7901 cells to cisplatin in RMPI-1640 plus 10% FBS by gradually increasing the cisplatin concentration until the cells acquired resistance to 50 μmol/L.

### Total RNA Extraction and Reverse Transcription Real-time Quantitative Polymerase Chain Reaction (RT-qPCR)

Total RNA was isolated from the tissues and cell lines using TRIzol reagent (Invitrogen, Eugene, OR, United States) according to the manufacturer’s protocol. For lncRNA and mRNA detection, RNA was reverse transcribed to cDNA using PrimeScript^TM^ RT reagent kit (Takara, Dalian, China). QPCR was performed using SYBR Premix Ex Taq^TM^ (Tli RNaseH Plus, Takara, Dalian, China) following the manufacturer’s instructions. GAPDH and UBC were used as internal controls. The PCR primers were synthesized by Biosune Biotechnology (Shanghai, China) and are listed in Supplementary Table S2. For miRNAs, the SYBR PrimeScript miRNA RT-qPCR Kit (Takara, Dalian, China) was used with U6 and RNU48 as internal controls. Experiments were performed on a CFX-96 Real-Time System (Bio-Rad, United States) and expression levels were calculated using the comparative quantification cycle (Cq) method. Each experiment was performed in triplicate, and the average Cq was recorded.

### Transfection

The HOTTIP coding sequence was amplified and cloned into pcDNA3.1 vector. The resulting construct, named pcDNA3.1-HOTTIP (Gene Chem, China), was used to overexpress HOTTIP. Si-HOTTIP, siRNA control, Atg5 siRNA, miR-216a-5p mimic, miR-216a-5p inhibitor, and control were all synthesized by RiboBio (Guangzhou, China). The siRNAs or miRNA inhibitor sequences are listed in Supplementary Table S3. Transfection was performed using Lipofectamine 2000 (Invitrogen) according to the manufacturer’s instructions.

### Xenograft Experiments

For *in vivo* experiments, SGC7901 cells (1 × 10^7^) transfected with the desired vector were subcutaneously injected into the flank area of 5-week-old male BALB/c athymic nude mice (*n* = 6 for each group). When the tumor volume reached 50–100 mm^3^, the xenograft tumor-bearing mice were intraperitoneally injected with cisplatin (10 μM/kg) every week. Tumors were measured and the tumor volume was calculated based on the following formula: Volume = 0.5 × length × width^2^. All mice were sacrificed 28 days after cell inoculation. All animal experiments were performed in compliance with the guidelines of the Animal Ethics Committee of Qilu Hospital of Shandong University.

### Cell Viability Assay

Chemosensitivity to the cells to cisplatin was quantified using the Cell Counting Kit-8 (CCK-8) (Best bio, China). Briefly, 5 × 10^3^ cells/well were seeded in 96-well plates. After transfection, the cells were treated with the indicated conditions. Then, 10 μL CCK-8 solution was added to the wells at the appropriate time points and the plates were incubated for 2 h at 37°C. Finally, the absorbance was measured at 450 nm using an ELISA plate reader (Thermo Scientific, United States) and the IC_50_ was calculated. Each experiment was performed in triplicate.

### Cell Apoptosis Assay

TUNEL assay was performed using the TUNEL BrightRed Apoptosis Detection Kit in accordance with the manufacturer’s instructions (Vazyme, Nanjing, China). Briefly, cells were plated in 24-well flat-bottom plates and incubated with 10 μM cisplatin for 24 h after transfection. Cells were fixed with 4% paraformaldehyde at 4°C for 30 min and permeabilized in 20 μg/ml proteinase K for 4 min before labeling with Bright Red labeling Mix for 1 h. Finally, the cells were incubated with DAPI (Beyotime, Beijing, China) for 5 min for nuclear staining, and sections were visualized with a laser scanning Olympus microscope (Tokyo, Japan).

For flow cytometric analysis, the transfected cells were incubated with 10 μM cisplatin for 24 h in plates. The cells were then collected and stained with PE Annexin V and 7-AAD (BD Bioscience, United States) in the dark for 15 min. Flow cytometrica nalysis was carried out using a FACSCanto II flow cytometer (BD Biosciences, Bedford, MA, United States).

### Cell Autophagy Assay

SGC7901/DDP cells were transfected with GFP-LC3 (Hanbio, Shanghai, China) to monitor autophagy following treatment with 10 μM cisplatin. The cells were then treated according to the designated treatment conditions. Finally, cells were fixed with 4% paraformaldehyde and GFP-LC3 punctate dot formation (corresponding to autophagosomes) was observed under a fluorescence microscope (Olympus, Tokyo, Japan).

### Luciferase Reporter Assay

A fragment of HOTTIP containing either the wild-type (wt) or mutated (mut) miR-216a-5p binding site was cloned into pMIR-REPORT luciferase vector (Invitrogen). MiR-216a-5p mimic or negative control was transfected into SGC7901/DDP cells with reporter plasmids using Lipofectamine 2000 (Invitrogen). The Dual-Luciferase Reporter Assay System (Promega, Madison, WI, United States) was used to detect the luciferase activity 48 h post-transfection and Renilla luciferase activity was normalize the firefly luciferase activity.

### Western Blot Assay

Total protein was extracted using RIPA buffer (Solarbio, Beijing, China) and the protein concentration was detected using a BCA protein kit (Beyotime, Beijing, China). Proteins were separated by sodium dodecyl sulfate (SDS)-polyacrylamide gel electrophoresis and transferred to a polyvinylidene difluoride (PVDF) membrane. The membranes were blocked with 5% fat-free milk, immunostained with primary antibodies at 4°C overnight followed by incubation with the secondary antibody for 1 h at room temperature and then detected using western chemiluminescent HRP substrate (Millipore, United States). The primary antibodies used were anti-Bcl-2 (1:1000; Cell Signaling Technology, United States), anti-Beclin1 (1:1000; Abcam, United Kingdom), anti-LC3I/II (1:1000; Cell Signaling Technology, United States), and anti-β-actin (1:5000; Cell Signaling Technology, United States).

### Statistical Analyses

Statistical analyses was performed using SPSS version 19.0 software, and figures were drawn using GraphPad Prism 5.0. The HOTTIP expression in GC tissue samples was non-normal distribution. And the comparisons between two groups according to Mann-Whitney test and the comparisons between multiple groups according to Kruskal-Wallis test. The significance of data in cell assays was compared by Unpaired student’s *t*-test (two-tailed). Kaplan-Meier analysis was performed using the log-rank test. The association between molecules was examined by Spearman correlation analysis. A receiver operating characteristic (ROC) curve was constructed using MedCalc Software v9.0 to estimate the diagnostic value. A *p*-value < 0.05 was considered statistically significant.

## Results

### HOTTIP Is Associated With Tumor Recurrence and Poor Survival in Patients With GC

**FIGURE 1:**
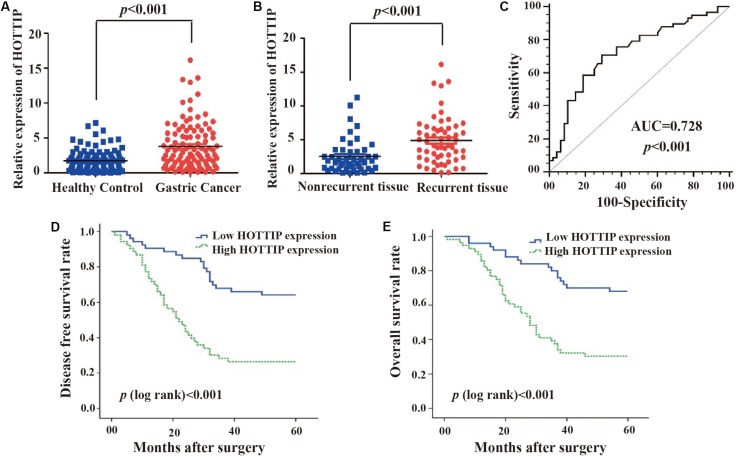
The clinical significance of HOTTIP in gastric cancer. **(A)** HOTTIP expression was analyzed by qRT-PCR in 106 paired gastric cancer (GC) and normal adjacent tissues. **(B)** HOTTIP expression was analyzed in chemotherapy-relapsed and non-relapsed tissue samples. **(C)** The ROC curve of HOTTIP for diagnosis of relapse in GC. **(D,E)** Kaplan-Meier curves for DFS **(D)** and OS **(E)** of GC patients with high versus low expression of HOTTIP.

To investigate the potential role of HOTTIP in GC, we first analyzed its expression profile in 106 paired human GC and adjacent normal tissues using RT-qPCR. As shown in Figure 1A, the expression of HOTTIP was much higher in the GC tissues than in the normal tissues (*p* < 0.001). In particular, HOTTIP expression was significantly upregulated in chemo-relapsed GC tissues (*p* < 0.001, Figure 1B). We then constructed ROC curves to analyze the diagnostic value of HOTTIP in GC recurrence. The area under the curve (AUC) for HOTTIP, indicative of the diagnostic value of the recurrence of GC, was 0.728, at the optimal cut-off value of 2.52, and the sensitivity and specificity were 70.7 and 70.8%, respectively, (*p* < 0.001, Figure 1C). Furthermore, we divided the GC patients into high and low HOTTIP expression groups according to the optimal cut-off value (2.52) and investigated the prognostic significance of HOTTIP expression. Kaplan-Meier analysis indicated that patients with low HOTTIP expression had better DFS (Figure 1D) and OS (Figure 1E) than those with high levels of HOTTIP (*p* < 0.001). Further, high level of HOTTIP was also correlated with tumor invasion depth (*p* < 0.001), lymph node metastasis (*p* = 0.0052), and TNM stage (*p* < 0.001, Table 1) in GC. Collectively, these data demonstrated that HOTTIP was associated with tumor recurrence and poor survival in GC.

**TABLE 1:** Correlation between lncRNA HOTTIP and clinicopathological parameters of gastric cancer (*n* = 106).

Features	Number of cases	HOTTIP expression levels	*P* value
Age (years)			
<61	52	3.140 (1.238–6.243)	0.2777
≥61	54	2.610 (1.065–5.048)	
Gender			
Female	48	3.290 (1.275–5.888)	0.5743
Male	58	2.580 (1.110–5.670)	
Tumer size (cm)			
<5	55	2.730 (1.210–5.340)	0.9647
≥5	51	3.240 (1.150–6.000)	
Pathological differention			
Well + moderate	31	2.400 (1.210–5.210)	0.6769
Poor	75	2.940 (1.210–6.020)	
Primary tumor site			
Proximal	22	2.445 (1.385–4.810)	0.6543
Antrum	47	3.470 (1.620–6.020)	
Body	31	2.470 (0.770–6.680)	
Multiple/diffuse	6	2.805 (0.588–5.478)	
Invasion depth			
T1-T3	45	1.150 (0.570–2.385)	<0.001
T4	61	5.020 (2.700–7.030)	
Lymph nodes metastasis			
No	13	1.210 (0.680–2.200)	0.0052
Yes	93	3.240 (1.545–6.045)	
TNM stage			
II	32	1.090 (0.500–2.278)	<0.001
III	74	4.005 (2.070–6.650)	
Tumor recurrence			
Yes	58	4.135 (2.278–6.650)	<0.001
No	48	1.750 (0.740–3.435)	

*Data presented as median (25%–75% interquartile range).*

### HOTTIP Is Upregulated in Cisplatin-resistant GC Cell Line

**FIGURE 2:**
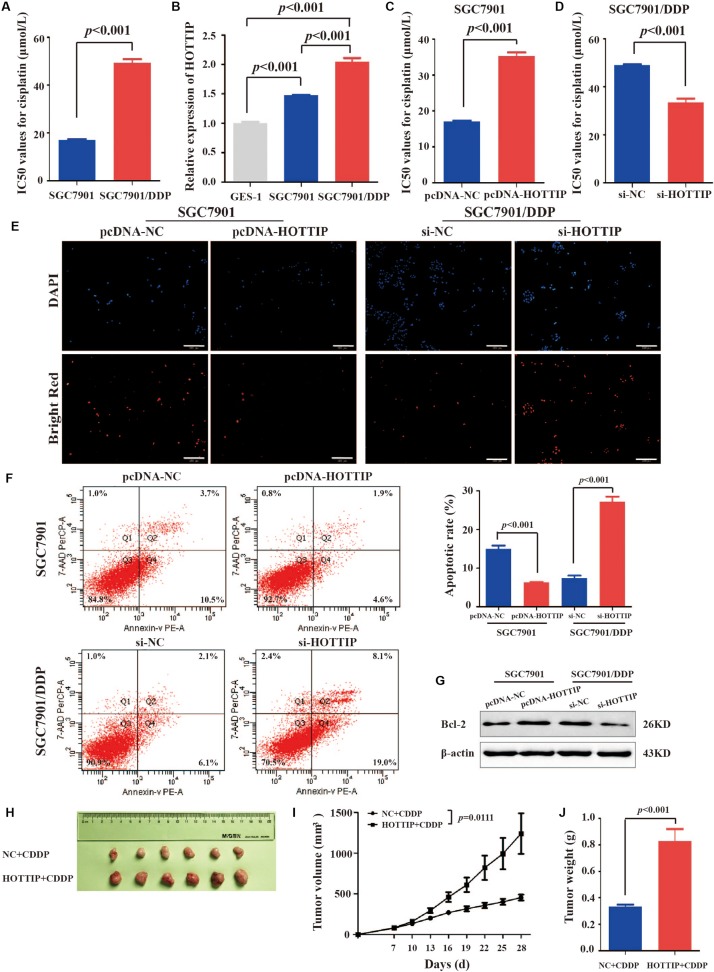
HOTTIP expression in GC cells and its expression in relation to chemoresistance and apoptosis of GC cells. **(A)** IC_50_ values of SGC7901 and SGC7901/DDP for cisplatin (IC_50_ = 16.96 and 49.22 μmol/L, respectively, *p* < 0.001). **(B)** HOTTIP expression was analyzed in GC and normal gastric epithelial cell lines. **(C)** The IC_50_ values of HOTTIP-overexpressing and control cells for cisplatin (IC_50_ = 35.21 and 17.10 μmol/L, respectively, *p* < 0.001). **(D)** The IC_50_ values of HOTTIP-silenced and control cells for cisplatin (IC_50_ = 33.52 and 49.08 μmol/L, respectively, *p* < 0.001). **(E,F)** TUNEL **(E)** and flow cytometry **(F)** Analysis the apoptosis in HOTTIP-overexpressing or HOTTIP-silenced, and control cells. Scale bar for TUNEL staining: 200 μm. **(G)** The relative expression of Bcl-2 protein in HOTTIP-overexpressing or HOTTIP-silenced, and control cells. **(H)** Images of tumor mass from each group (*n* = 6) on the 28th day. **(I,J)** The volumes and weights of tumors derived from HOTTIP overexpression cells were significantly larger than those from negative control cells.

We tested cisplatin resistance of GC cell lines using the CCK-8 assay. The results showed that SGC7901/DDP had higher IC_50_ than SGC7901 (IC_50_ = 49.22 and 16.96 μmol/L, respectively, *p* < 0.001, Figure 2A). To investigate the role of HOTTIP in cisplatin resistance of GC cells, we performed RT-qPCR analysis to examine the expression of HOTTIP in several cell lines, including GC (SGC7901 and SGC7901/DDP) and the human normal gastric epithelial cell lines (GES-1). SGC7901/DDP expressed higher level of HOTTIP than SGC7901 (*p* < 0.001), and these two GC cell lines showed 1.473- and 2.042-fold upregulation of HOTTIP compared to GES-1 cells (Figure 2B). Thus, HOTTIP overexpression potentially played a role in cisplatin resistance in the GC cells.

### HOTTIP Regulates Chemoresistance in GC Cells

To explore the effect of HOTTIP expressionon the biological properties of the GC cells, we overexpressed or silenced HOTTIP in GC cells and determined their cisplatin resistance and apoptotic abilities. Firstly, HOTTIP overexpression efficiency in SGC7901 cells (Supplementary Figure S1A) and HOTTIP silencing efficiencies in SGC7901/DDP cells (Supplementary Figure S1B) were verified to be significant. CCK-8 assay results revealed that HOTTIP overexpression notably increased cisplatin resistance of SGC7901 cells compared to control cells (IC_50_ = 35.21 and 17.10 μmol/L, respectively, *p* < 0.001, Figure 2C) while silencing of HOTTIP significantly inhibited cisplatin resistance of SGC7901/DDP cells compared to cells transfected with the empty vector (IC_50_ = 49.08 and 33.52 μmol/L, respectively, *p* < 0.001, Figure 2D). The results of TUNEL and PE Annexin V/7-AAD apoptosis analyses revealed that overexpression of HOTTIP in SGC7901 cells significantly decreased cell apoptosis while silencing of HOTTIP significantly increased cell apoptosis (Figures 2E,F). Western blotting results (Figure 2G) indicated that the expression level of the anti-apoptotic protein, Bcl-2, was increased following overexpression of HOTTIP in SGC7901 cells and decreased following silencing of HOTTIP in SGC7901/DDP cells. Together, these results indicate that HOTTIP regulates cisplatin resistance and apoptosis *in vitro*.

To evaluate the effect of HOTTIP on chemoresistance *in vivo*, we established a xenograft tumor model using SGC7901 cells transfected with pcDNA-HOTTIP in nude mice. As shown in Figures 2H–J, the HOTTIP overexpression group displayed larger tumor size and weight than those in the NC group, which was consistent with the *in vitro* observations.

### Silencing of HOTTIP Inhibits Chemoresistance of GC Cells by Promoting Autophagy

**FIGURE 3:**
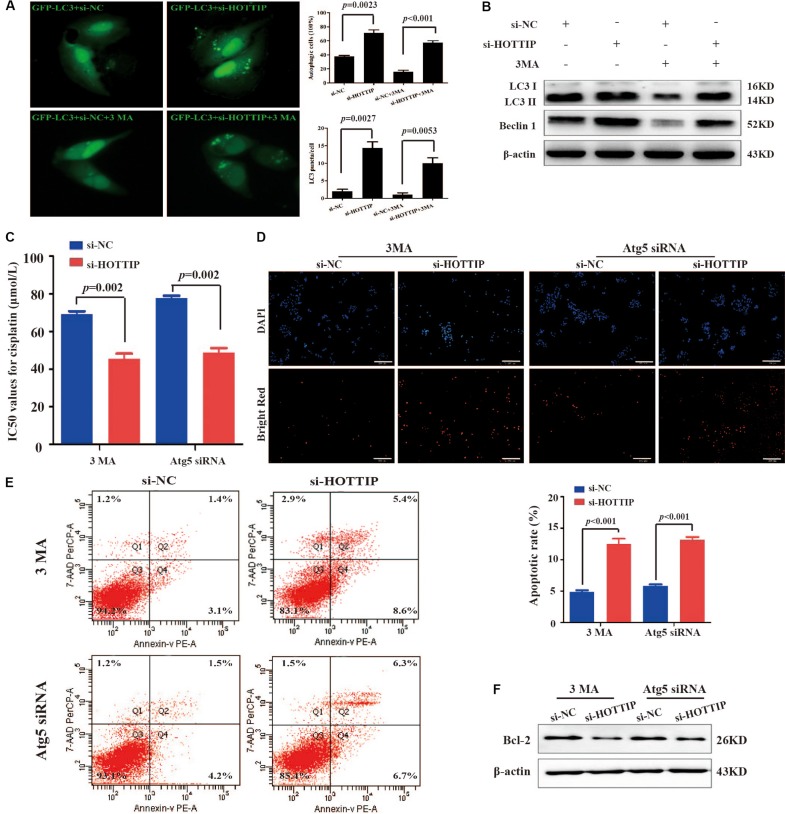
The relationship between HOTTIP expression and autophagy in GC cells. **(A)** Accumulation of GFP-LC3 puncta in SGC7901/DDP cells co-transfected with si-NC, si-HOTTIP, si-NC + 3MA, or si-HOTTIP + 3MA was analyzed by fluorescence microscopy. Scale bar = 25 μm. **(B)** Relative expression of autophagy-related proteins in SGC7901/DDP following transfection of si-NC, si-HOTTIP, si-NC + 3MA, or si-HOTTIP + 3MA, respectively. **(C)** IC_50_ values for cisplatin of HOTTIP-silenced cells and control cells pretreated with 3MA or Atg5 siRNA. **(D,E)** TUNEL **(D)** and flow cytometry **(E)** analysis of apoptosis in HOTTIP-silenced cells and control cells pretreated with 3MA or Atg5 siRNA. Scale bar for TUNEL staining: 200 μm. **(F)** The relative expression of Bcl-2 protein in HOTTIP-silenced cells and control cells pretreated with 3MA or Atg5 siRNA. Data are presented as mean ± standard error of mean (SEM).

Depending on different tumor types and therapeutic features, autophagy can play either a pro-survival or a pro-death role in response to chemotherapy drugs (Fulda, 2017). Therefore, we next explored whether HOTTIP affected chemoresistance in GC cells by regulating autophagy. GFP-LC3, a GFP-tagged specific marker of autophagosomes (Kabeya et al., 2000), was transfected into SGC7901/DDP cells to facilitate the visualization of autophagy. First, the number of GFP-LC3 puncta-positive cells and LC3 puncta/cell in HOTTIP-silenced cells was analyzed compared to the control cells (Figure 3A). We then treated the SGC7901/DDP cells with 1 mM of 3MA (an inhibitor of autophagy; Liu et al., 2011) to further verify our results. We found that HOTTIP silencing reversed the effect of 3MA on autophagy of GC cells (Figure 3A). As shown in Figure 3B, western blot assay also demonstrated that 3MA could significantly inhibit the expression of autophagy-related proteins (LC3-II and Beclin1), while silencing of HOTTIP abrogated the 3MA-mediated autophagy inhibitory effect. Taken together, these data suggest that silencing of HOTTIP activates autophagy in GC.

Furthermore, 3MA or siRNA-mediated depletion of Atg5 (a protein required for autophagosome formation; Mizushima et al., 2001) was used to inhibit autophagy to investigate whether HOTTIP affects cisplatin resistance by regulating autophagy in GC. CCK-8 and apoptosis assay results showed that the IC_50_ of SGC7901/DDP cells was significantly increased (IC_50_: si-NC + 3MA vs. si-HOTTIP + 3MA = 69.13 μmol/L vs. 45.39 μmol/L, *p* = 0.0020; si-NC + Atg5 siRNA vs. si-HOTTIP + Atg5 siRNA = 77.77 μmol/L vs. 48.96 μmol/L, *p* = 0.0004) and that apoptosis was markedly decreased following 3MA or Atg5 siRNA treatment. Further, these effects were partially reversed by HOTTIP knockdown (Figures 3C–F). All these results demonstrated that knockdown of HOTTIP inhibits chemoresistance of SGC7901/DDP by promoting autophagy.

### HOTTIP Regulates Chemoresistance in GC Cells by Sponging MiR-216a-5p

**FIGURE 4:**
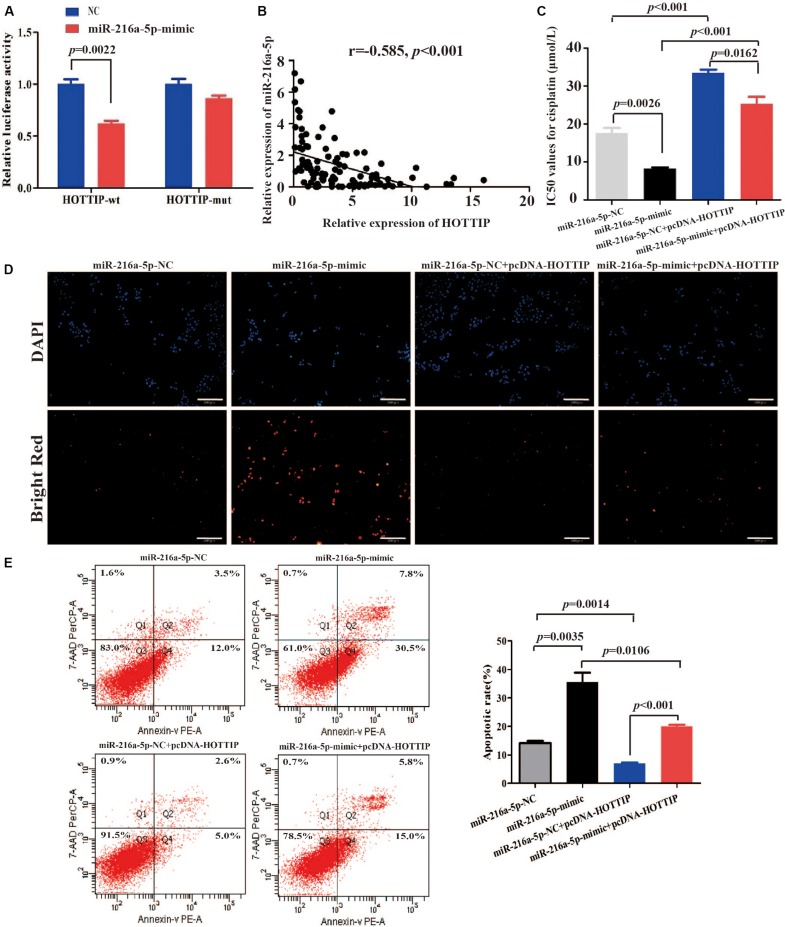
HOTTIP directly interacts with miR-216a-5p to play key roles in GC. **(A)** Relative luciferase activity of HOTTIP containing wild-type or mutant miR-216a-5p binding site or controls. **(B)** There was a significant negative correlation between lncRNA HOTTIP and miR-216a-5p expression in GC tissues. **(C)** The IC_50_ values for cisplatin of SGC7901 following transfection of miR-216a-5p NC, miR-216a-5p mimic, miR-216a-5p NC + pcDNA-HOTTIP or miR-216a-5p mimic + pcDNA-HOTTIP, respectively (IC_50_: miR-216a-5p NC vs. miR-216a-5p mimic = 17.61 μmol/L vs. 8.247 μmol/L, respectively, *p* = 0.0026; miR-216a-5p NC + pcDNA-HOTTIP vs. miR-216a-5p mimic + pcDNA-HOTTIP = 33.43 μmol/L vs. 25.30 μmol/L, respectively, *p* = 0.0162). **(D,E)** TUNEL **(D)** and flow cytometry **(E)** analysis of apoptosis in SGC7901 cells following transfection with miR-216a-5p NC, miR-216a-5p mimic, miR-216a-5p NC + pcDNA-HOTTIP or miR-216a-5p mimic + pcDNA-HOTTIP, respectively. Scale bar for TUNEL staining: 200 μm.

Long non-coding RNA can act as aceRNA to sequester miRNAs, thereby liberating the corresponding miRNA-targeted transcripts (Qu et al., 2016). It has been reported that miR-216a-5p has a binding site for HOTTIP (Sun et al., 2018; Yang et al., 2018). Thus, we sought to explore the mechanism of miR-216a-5p regulation by HOTTIP. We performed a dual-luciferase reporter assay to examine the HOTTIP putative binding with miR-216a-5p. As shown in Figure 4A, luciferase activity was significantly decreased after co-transfection with HOTTIP-wt and miR-216a-5p mimic (*p* = 0.0022), while the miR-216a-5p mimic had no significant influence on the luciferase activity following co-transfection with HOTTIP-mut plasmid (*p* > 0.05). Subsequently, we analyzed the correlation between expression of HOTTIP and miR-216a-5p in GC tissues, and found a significant negative correlation (*r* = -0.585, *p* < 0.001; Figure 4B).

We found that overexpression of miR-216a-5p significantly inhibited cisplatin resistance (IC_50_ = 17.61 and 8.247 μmol/L, respectively, *p* = 0.0026; Figure 4C) and promoted cell apoptosis (Figures 4D,E). The co-transfection of pcDNA-HOTTIP and miR-216a-5p mimic significantly decreased cisplatin resistance (IC_50_ = 33.43 and 25.30 μmol/L, respectively, *p* = 0.0162) and enhanced cell apoptosis compared to co-transfection of pcDNA-HOTTIP and miR-216a-5p NC in SGC7901 cells (Figures 4C–E). Furthermore, we found that ectopic expression of HOTTIP did not affect cisplatin resistance, apoptosis, or autophagy ability in SGC7901 compared to cells transfected with the empty vector after blocking miR-216a-5p (Supplementary Figures S2A–D). These results indicated that HOTTIP regulates cisplatin resistance and apoptosis of GC cells by sponging miR-216a-5p.

### MiR-216a-5p Interacts With Bcl-2 to Regulate Cellular Activity

**FIGURE 5:**
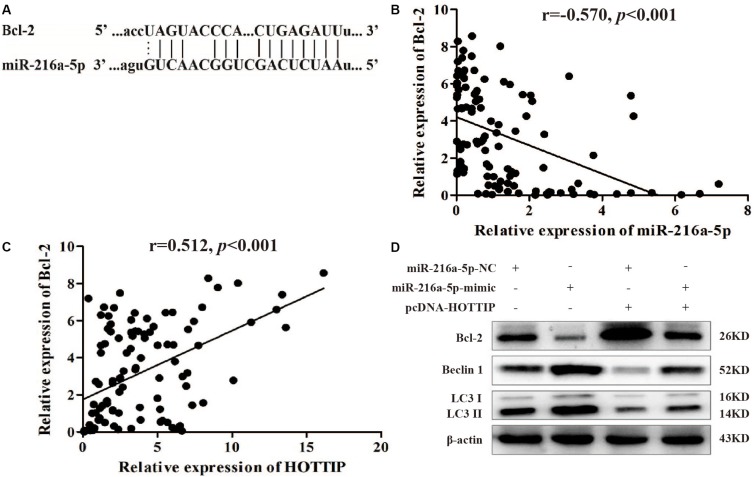
MiR-216a-5p interacts with Bcl-2 to regulate the biological activity of GC cells. **(A)** Bcl-2 binding sites in miR-216a-5p were predicted using starBase v2.0. **(B)** There was a significant negative correlation between miR-216a-5p and Bcl-2 expression in GC tissues. **(C)** There was a positive correlation between HOTTIP and Bcl-2 expression in GC tissues. **(D)** Relative expression of Bcl-2 and autophagy-related proteins in SGC7901 following transfection of miR-216a-5p NC, miR-216a-5p mimic, miR-216a-5p NC + pcDNA-HOTTIP, or miR-216a-5p mimic + pcDNA-HOTTIP, respectively.

We focused on Bcl-2 as a target gene of miR-216a-5p (predicted by starBase v2.0)^1^ because of its role in regulating autophagy and apoptosis (Figure 5A). Spearman test demonstrated a significant negative correlation between miR-216a-5p and Bcl-2 expression in GC tissues (*r* = −0.570, *p* < 0.001, Figure 5B), which was consistent with the prediction. The Spearman test also showed that there was a positive correlation between HOTTIP and Bcl-2 expression in GC tissues (*r* = 0.512, *p* < 0.001, Figure 5C), indicating that HOTTIP may function as a ceRNA to miR-216a-5p to promote Bcl-2 expression.

Western blot analysis showed that upregulation of miR-216a-5p markedly reduced the expression of Bcl-2 at the protein level and accelerated the expression of LC3-II and Beclin1. The overexpression of HOTTIP significantly alleviated these changes (Figure 5D). Based on these findings, we propose that HOTTIP/miR-216a-5p/Bcl-2/Beclin1 may be the core axis regulating apoptosis, autophagy, and chemoresistance in GC. Thus, HOTTIPrepresents a promising therapeutic target for GC treatment in the future.

## Discussion

Cisplatin is an effective and standard chemotherapeutic drug used in GC (Einhorn and Donohue, 1977), and resistance to cisplatin is closely associated with poor prognosis. Therefore, determination of the molecular mechanism underlying cisplatin resistance is urgently needed for GC therapy. Accumulating evidence indicates that abnormal expression of lncRNAs is closely correlated with the drug resistance of cancer cells through various mechanisms, such as modulating drug efflux, DNA damage repair, and apoptosis (Chen et al., 2017), including cisplatin resistance (Hu et al., 2018). Previous studies have shown an important role of the lncRNA, HOTTIP, in resistance to anticancer drugs (Li et al., 2016; Zhang et al., 2017; Sun et al., 2018). For example, HOTTIP was shown to be involved in the development of chemoresistance in lung adenocarcinoma by regulating the AKT signaling pathway (Zhang et al., 2017). In osteosarcoma, HOTTIP overexpression was also found to be associated with poor response to chemotherapy by activating the Wnt/β-catenin pathway (Li et al., 2016). However, the functional role of HOTTIP in chemoresistance in GC is largely unknown. In this study, we report for the first time that HOTTIP is significantly upregulated in chemotherapy-relapsed GC, and its expression level is inversely correlated with cisplatin sensitivity of GC cells. Functionally, HOTTIP negatively regulates apoptosis and autophagy and promotes chemoresistance in GC cells. Further investigations revealed that by sponging miR-216a-5p, HOTTIP enhanced the cisplatin resistance of GC cells by regulating the Bcl-2/Beclin1/autophagy pathway.

The role of autophagy in regulating tumor chemosensitivity has been reported extensively recently. Zhou et al. (2017) found that tunicamycin induced endoplasmic reticulum stress (ERS) and consequently autophagy and apoptosis in human esophageal cancer cells, which rendered them more sensitive to cisplatin. Xu et al. (2018) demonstrated that Rab5a enhanced cisplatin resistance through inhibition of autophagy in GC cells. Our experiments indicated that the autophagy inhibitor, 3MA, increased cisplatin resistance of GC cells, and silencing HOTTIP reduced cisplatin resistance by promoting autophagy. Therefore, knockdown of HOTTIP could promote cell death of GC cells by inducing pro-death autophagy, which provides new insights into the biological mechanism of cisplatin resistance in GC.

Recent studies have revealed that lncRNAs play important and powerful roles in regulating gene activity (Nagano and Fraser, 2011). The competing endogenous RNAs (ceRNAs) hypothesis and the role of lncRNAs as ceRNAs has received considerable attention recently (Thomson and Dinger, 2016). For example, lncRNA SNHG6 functions as a ceRNA by regulating the expression of miR-101-3p in GC (Yan et al., 2017), and lncRNA MALAT1 increases the chemoresistance of GC by regulating miRNA-23b-3p (Hu et al., 2017). The clustered miR-216 family has recently been reported to play a tumor-suppressive role in human cancer and HOTTIP could competitively sequester miR-216a in small cell lung cancer and prostate cancer (Yonemori et al., 2017; Sun et al., 2018; Yang et al., 2018). To investigate whether HOTTIP acts as a ceRNA by sponging miR-216a-5p in GC cells, we performed luciferase reporter assay and found that miR-216a-5p directly bound to HOTTIP. We further explored the function of HOTTIP/miR-216a-5p in GC cells and found that overexpression of miR-216a-5p increased apoptosis and autophagy and promoted cisplatin sensitivity of SGC7901. HOTTIP reversed these effects caused by miR-216a-5p.

It is reported that lncRNAs may regulate their neighboring protein-coding genes in a *cis*-manner (Guil and Esteller, 2012). HOTTIP is located on the 5′ end of the HOXA gene cluster. Wang et al. (2011) demonstrated that HOTTIP primarily targets WDR5/MLL complexes across HOXA by directly binding to the adaptor protein WDR5, leading to histone H3 lysine 4 trimethylation and gene transcription of several 5′ HOXA genes. Cheng et al. (2015) reported a positive correlation between HOTTIP and HOXA expression in pancreatic cancer, including HOXA1, HOXA9, HOXA10, and HOXA11. In addition, Chang et al. (2016) showed that HOTTIP and HOXA13 are involved in the tumorigenesis and progression of GC. Several studies have shown that aberrant expression of HOXA genes is associated with biological characteristics in GC (Sentani et al., 2012; Han et al., 2013; Bai et al., 2014). HOTTIP may be critical in regulating HOXA genes in GC, but the underlying mechanism remains unknown and requires further study.

MicroRNAs can bind to the 3′ UTR of target genes and thereby block the translation of RNA (Park and Kim, 2019). In our study, we identified Bcl-2 as the downstream effector of miR-216a-5p using the starBase database and demonstrateda negative correlation between miR-216a-5p and Bcl-2 expression in GC tissues. The role of Bcl-2 in chemoresistance by regulating autophagy has been reported previously (Li et al., 2018). Bcl-2 has hydrophobic grooves that can accommodate the BH3 structure domain of Beclin1, an important regulator of autophagy initiation (Zhang et al., 2015). When Bcl-2 binds to Beclin1, it forms the Bcl-2-Beclin1 complex and inhibits Beclin1-dependent autophagy (Pattingre et al., 2005). In our study, overexpression of miR-216a-5p inhibited the expression of Bcl-2, relieving the inhibitory effect on Beclin1, and thus initiating autophagy, which was consistent with the effects induced by downregulation of HOTTIP. Taken together these data suggest that HOTTIP sponges miR-216a-5p to promote the expression of Bcl-2, thus increasing the formation of the Bcl-2-Beclin1 complex, and finally decreasing autophagy-related cell death, which leads to cisplatin resistance in GC.

In conclusion, our work identified that HOTTIP inhibits autophagy and promotes cisplatin resistance in GC. These findings may help understand the potential molecular mechanisms underlying resistance to chemotherapeutic drugs, and may provide evidence for a novel combined therapeutic strategy to overcome drug resistance in GC.

## Data Availability Statement

The data that support the findings of this study are available from the corresponding author upon reasonable request.

## Ethics Statement

The studies involving human participants were reviewed and approved by The Ethics Committee of the Qilu Hospital of Shandong University. The patients/participants provided their written informed consent to participate in this study.

## Author Contributions

XZa and YiZ conducted the study design. RZ, YanZ, YY, and YS performed experiments and analyzed the data. RZ, XZe, AQ, and YapZ collected the samples. RZ, XZa, YY, and YU drafted the manuscript. All the authors reviewed and approved the final manuscript.

## Conflict of Interest

The authors declare that the research was conducted in the absence of any commercial or financial relationships that could be construed as a potential conflict of interest.

## Footnotes

1 http://starbase.sysu.edu.cn/

## Supplementary Material

TABLE S1 The clinicopathological parameters of gastric cancer patients (*n* = 106).

TABLE S2 The sequences of RT-qPCR primer.

TABLE S3 The sequences of the siRNA primers.

FIGURE S1 HOTTIP overexpression or silencing efficiency. **(A)** HOTTIP overexpression efficiency in SGC7901 cells. **(B)** HOTTIP silencing efficiency in SGC7901/DDP cells.

FIGURE S2 HOTTIP directly interacts with miR-216a-5p to play key roles in gastric cancer. **(A)** The IC50 values for cisplatin of SGC7901 following transfection of anti-miR-NC + pcDNA-HOTTIP or anti-miR-216a-5p + pcDNA-HOTTIP, respectively (IC_50_ = 38.92 μmol/l vs. 37.50 μmol/l, respectively, *p* = 0.6804). **(B,C)** TUNEL **(B)** and flow cytometry **(C)** apotosis analysis showed the apoptosis ability in SGC7901 following transfection of anti-miR-NC + pcDNA-HOTTIP or anti-miR-216a-5p + pcDNA-HOTTIP, respectively. Scale bar for TUNEL staining: 200 μm. **(D)** The relative expression of Bcl-2 protein in SGC7901 following transfection of anti-miR-NC + pcDNA-HOTTIP or anti-miR-216a-5p + pcDNA-HOTTIP, respectively.
