# Supplementary material for: HOTTIP Predicts Poor Survival in Gastric Cancer Patients and Contributes to Cisplatin Resistance by Sponging miR-216a-5p
Source: Front Cell Dev Biol. 2020 May 8;8:348. doi: 10.3389/fcell.2020.00348 (PMC7225723; doi:10.3389/fcell.2020.00348)
Supplement: TABLE S1 — The clinicopathological parameters of gastric cancer patients (n = 106). [file Table_1.docx]

**Table S1**. The clinicopathological parameters of gastric cancer patients (n=106)

| **Number** | **Age**  **(years)** | **Gender** | **Invasion depth** | **Lymph nodes metastasis** | **TNM stage** | **Primary tumor site** | **Tumer size(cm)** | **Pathological differention** | **Retime (month)** | **Ostime (month)** |
| --- | --- | --- | --- | --- | --- | --- | --- | --- | --- | --- |
| **1** | **72** | **male** | **T2** | **N1** | **IIA** | **Proximal** | **4** | **well** | **24** | **28** |
| **2** | **61** | **female** | **T2** | **N1** | **IIA** | **Antrum** | **8.5** | **poor** | **no** | **survival** |
| **3** | **66** | **male** | **T3** | **N2** | **IIIA** | **Antrum** | **3** | **well** | **30** | **survival** |
| **4** | **65** | **female** | **T4b** | **N3a** | **IIIC** | **Antrum** | **8** | **poor** | **no** | **survival** |
| **5** | **55** | **female** | **T3** | **N3a** | **IIIB** | **Proximal** | **0.8** | **well** | **32** | **35** |
| **6** | **57** | **male** | **T4b** | **N2** | **IIIB** | **Antrum** | **7.5** | **well** | **15** | **survival** |
| **7** | **45** | **male** | **T2** | **N2** | **IIB** | **Proximal** | **3.5** | **moderate** | **no** | **survival** |
| **8** | **30** | **male** | **T3** | **N2** | **IIIA** | **Proximal** | **5.6** | **poor** | **30** | **34** |
| **9** | **69** | **male** | **T1** | **N2** | **IIA** | **Body** | **4.3** | **poor** | **no** | **survival** |
| **10** | **54** | **female** | **T2** | **N2** | **IIB** | **Antrum** | **3.1** | **poor** | **no** | **survival** |
| **11** | **69** | **male** | **T4a** | **N0** | **IIB** | **Antrum** | **3.5** | **poor** | **6** | **8** |
| **12** | **62** | **female** | **T3** | **N2** | **IIIA** | **Proximal** | **1.2** | **poor** | **10** | **20** |
| **13** | **45** | **male** | **T3** | **N0** | **IIA** | **Body** | **7.5** | **well** | **no** | **survival** |
| **14** | **68** | **male** | **T4b** | **N1** | **IIIB** | **Proximal** | **1.5** | **poor** | **no** | **survival** |
| **15** | **59** | **male** | **T3** | **N1** | **IIB** | **Body** | **6** | **poor** | **49** | **54** |
| **16** | **60** | **female** | **T3** | **N3a** | **IIIB** | **Body** | **8.4** | **poor** | **no** | **survival** |
| **17** | **62** | **male** | **T2** | **N2** | **IIB** | **Body** | **7.7** | **moderate** | **32** | **40** |
| **18** | **67** | **female** | **T4b** | **N1** | **IIIB** | **Antrum** | **2.5** | **well** | **no** | **survival** |
| **19** | **69** | **male** | **T2** | **N2** | **IIB** | **Multiple** | **1.2** | **moderate** | **17** | **20** |
| **20** | **51** | **male** | **T4a** | **N3a** | **IIIB** | **Proximal** | **4** | **poor** | **no** | **survival** |
| **21** | **43** | **female** | **T4b** | **N0** | **IIIA** | **Antrum** | **4.8** | **poor** | **32** | **38** |
| **22** | **65** | **male** | **T4a** | **N0** | **IIB** | **Proximal** | **7** | **poor** | **no** | **survival** |
| **23** | **56** | **male** | **T3** | **N3a** | **IIIB** | **Multiple** | **8** | **poor** | **no** | **survival** |
| **24** | **48** | **female** | **T2** | **N1** | **IIA** | **Body** | **3** | **poor** | **5** | **8** |
| **25** | **72** | **male** | **T4a** | **N3b** | **IIIC** | **Proximal** | **10** | **poor** | **22** | **28** |
| **26** | **55** | **male** | **T4a** | **N3a** | **IIIB** | **Antrum** | **3.5** | **poor** | **no** | **survival** |
| **27** | **74** | **female** | **T4b** | **N1** | **IIIB** | **Antrum** | **4.5** | **poor** | **no** | **survival** |
| **28** | **66** | **female** | **T3** | **N0** | **IIA** | **Body** | **4.3** | **moderate** | **34** | **37** |
| **29** | **61** | **female** | **T3** | **N0** | **IIA** | **Antrum** | **7.5** | **poor** | **no** | **survival** |
| **30** | **63** | **male** | **T4a** | **N0** | **IIB** | **Body** | **10.2** | **poor** | **11** | **16** |
| **31** | **39** | **male** | **T2** | **N3a** | **IIIA** | **Body** | **1.3** | **poor** | **no** | **survival** |
| **32** | **58** | **male** | **T4b** | **N3a** | **IIIC** | **Antrum** | **1.9** | **poor** | **1** | **1** |
| **33** | **68** | **male** | **T4a** | **N0** | **IIB** | **Antrum** | **3** | **poor** | **no** | **survival** |
| **34** | **67** | **male** | **T3** | **N3a** | **IIIB** | **Body** | **2** | **poor** | **7** | **15** |
| **35** | **32** | **male** | **T2** | **N1** | **IIA** | **Body** | **6.5** | **moderate** | **no** | **survival** |
| **36** | **72** | **female** | **T4a** | **N3b** | **IIIC** | **Body** | **5** | **well** | **no** | **survival** |
| **37** | **67** | **female** | **T4a** | **N3a** | **IIIB** | **Proximal** | **3.7** | **poor** | **10** | **19** |
| **38** | **57** | **male** | **T4a** | **N3b** | **IIIC** | **Proximal** | **4** | **poor** | **39** | **46** |
| **39** | **59** | **female** | **T2** | **N3b** | **IIIB** | **Antrum** | **3.4** | **poor** | **no** | **survival** |
| **40** | **60** | **male** | **T4a** | **N3a** | **IIIB** | **Body** | **8** | **well** | **21** | **23** |
| **41** | **69** | **female** | **T4b** | **N2** | **IIIB** | **Body** | **5.4** | **moderate** | **7** | **10** |
| **42** | **70** | **male** | **T2** | **N2** | **IIB** | **Body** | **5.8** | **poor** | **no** | **survival** |
| **43** | **55** | **female** | **T4b** | **N0** | **IIIA** | **Antrum** | **2.9** | **poor** | **no** | **survival** |
| **44** | **74** | **female** | **T4a** | **N1** | **IIIA** | **Proximal** | **6** | **moderate** | **no** | **survival** |
| **45** | **75** | **male** | **T3** | **N1** | **IIB** | **Body** | **6.5** | **poor** | **no** | **survival** |
| **46** | **45** | **female** | **T2** | **N2** | **IIB** | **Antrum** | **7** | **poor** | **38** | **survival** |
| **47** | **67** | **female** | **T4b** | **N3b** | **IIIC** | **Antrum** | **1** | **poor** | **no** | **survival** |
| **48** | **29** | **male** | **T3** | **N0** | **IIA** | **Proximal** | **2.8** | **poor** | **no** | **survival** |
| **49** | **65** | **female** | **T2** | **N2** | **IIB** | **Antrum** | **2.4** | **moderate** | **no** | **survival** |
| **50** | **74** | **female** | **T2** | **N2** | **IIB** | **Body** | **3** | **moderate** | **no** | **survival** |
| **51** | **45** | **female** | **T4b** | **N3a** | **IIIC** | **Antrum** | **4** | **poor** | **no** | **survival** |
| **52** | **66** | **female** | **T2** | **N1** | **IIA** | **Antrum** | **4.5** | **poor** | **29** | **37** |
| **53** | **75** | **female** | **T2** | **N1** | **IIA** | **Antrum** | **6.2** | **poor** | **23** | **24** |
| **54** | **35** | **female** | **T4b** | **N3a** | **IIIC** | **Antrum** | **2.5** | **poor** | **12** | **15** |
| **55** | **70** | **female** | **T3** | **N3a** | **IIIB** | **Body** | **2.5** | **well** | **27** | **30** |
| **56** | **57** | **male** | **T3** | **N0** | **IIA** | **diffuse** | **6** | **moderate** | **no** | **survival** |
| **57** | **56** | **male** | **T3** | **N3b** | **IIIC** | **Body** | **0.8** | **poor** | **no** | **survival** |
| **58** | **62** | **female** | **T3** | **N1** | **IIB** | **Body** | **7** | **poor** | **no** | **survival** |
| **59** | **58** | **female** | **T3** | **N2** | **IIIA** | **Antrum** | **2** | **poor** | **23** | **25** |
| **60** | **55** | **male** | **T4a** | **N2** | **IIIA** | **Antrum** | **4** | **poor** | **21** | **27** |
| **61** | **53** | **male** | **T3** | **N1** | **IIB** | **Antrum** | **8** | **moderate** | **no** | **survival** |
| **62** | **74** | **female** | **T4b** | **N0** | **IIIA** | **Proximal** | **3** | **poor** | **no** | **survival** |
| **63** | **58** | **male** | **T4b** | **N2** | **IIIB** | **Antrum** | **9.8** | **well** | **5** | **8** |
| **64** | **56** | **female** | **T4a** | **N0** | **IIB** | **Antrum** | **7.3** | **moderate** | **no** | **survival** |
| **65** | **74** | **male** | **T4b** | **N3a** | **IIIC** | **Body** | **3** | **poor** | **31** | **31** |
| **66** | **56** | **male** | **T4b** | **N3a** | **IIIC** | **Antrum** | **5.5** | **poor** | **17** | **20** |
| **67** | **67** | **male** | **T4b** | **N2** | **IIIB** | **Antrum** | **4.5** | **poor** | **no** | **survival** |
| **68** | **75** | **female** | **T3** | **N1** | **IIB** | **Antrum** | **4.2** | **poor** | **no** | **survival** |
| **69** | **67** | **male** | **T4b** | **N3a** | **IIIC** | **Body** | **9** | **poor** | **32** | **35** |
| **70** | **68** | **male** | **T2** | **N3a** | **IIIA** | **Antrum** | **8.7** | **poor** | **no** | **survival** |
| **71** | **56** | **male** | **T4b** | **N3a** | **IIIC** | **Antrum** | **3.5** | **poor** | **17** | **19** |
| **72** | **67** | **male** | **T4b** | **N1** | **IIIB** | **Body** | **7.9** | **poor** | **no** | **survival** |
| **73** | **67** | **male** | **T4a** | **N3a** | **IIIB** | **Antrum** | **7.5** | **poor** | **no** | **survival** |
| **74** | **45** | **male** | **T2** | **N1** | **IIA** | **Antrum** | **4.5** | **well** | **21** | **25** |
| **75** | **66** | **male** | **T4b** | **N3a** | **IIIC** | **Multiple** | **3** | **poor** | **35** | **37** |
| **76** | **66** | **male** | **T4a** | **N3a** | **IIIB** | **Body** | **6.5** | **poor** | **no** | **survival** |
| **77** | **44** | **female** | **T3** | **N3b** | **IIIC** | **Antrum** | **6** | **poor** | **no** | **survival** |
| **78** | **46** | **female** | **T4a** | **N3a** | **IIIB** | **Antrum** | **5** | **moderate** | **32** | **38** |
| **79** | **48** | **male** | **T4b** | **N3b** | **IIIC** | **Body** | **3** | **poor** | **24** | **30** |
| **80** | **69** | **male** | **T4a** | **N3a** | **IIIB** | **Antrum** | **5** | **poor** | **26** | **28** |
| **81** | **59** | **male** | **T4a** | **N3b** | **IIIC** | **Antrum** | **2.8** | **poor** | **18** | **18** |
| **82** | **61** | **female** | **T4b** | **N2** | **IIIB** | **Body** | **2.5** | **well** | **17** | **21** |
| **83** | **55** | **male** | **T4b** | **N3b** | **IIIC** | **Body** | **7** | **poor** | **no** | **survival** |
| **84** | **54** | **female** | **T4b** | **N3a** | **IIIC** | **Proximal** | **7.5** | **poor** | **10** | **12** |
| **85** | **67** | **female** | **T4b** | **N3b** | **IIIC** | **Antrum** | **8** | **poor** | **20** | **25** |
| **86** | **65** | **male** | **T3** | **N3a** | **IIIB** | **Multiple** | **8** | **poor** | **11** | **13** |
| **87** | **56** | **female** | **T4b** | **N3a** | **IIIC** | **Antrum** | **3.5** | **poor** | **3** | **5** |
| **88** | **34** | **female** | **T4b** | **N2** | **IIIB** | **Proximal** | **8.5** | **poor** | **no** | **survival** |
| **89** | **57** | **male** | **T4b** | **N2** | **IIIB** | **Body** | **3** | **moderate** | **14** | **17** |
| **90** | **63** | **female** | **T4a** | **N3b** | **IIIC** | **Antrum** | **9** | **poor** | **6** | **11** |
| **91** | **47** | **female** | **T4a** | **N3a** | **IIIB** | **Body** | **2.8** | **poor** | **3** | **6** |
| **92** | **52** | **male** | **T4b** | **N2** | **IIIB** | **Body** | **3.5** | **well** | **13** | **15** |
| **93** | **62** | **male** | **T4a** | **N3b** | **IIIC** | **Antrum** | **4.5** | **well** | **8** | **12** |
| **94** | **71** | **female** | **T3** | **N2** | **IIIA** | **Proximal** | **6.5** | **poor** | **no** | **survival** |
| **95** | **73** | **male** | **T4a** | **N2** | **IIIA** | **Proximal** | **6** | **moderate** | **no** | **survival** |
| **96** | **55** | **male** | **T4b** | **N3a** | **IIIC** | **Antrum** | **4.3** | **poor** | **12** | **18** |
| **97** | **74** | **female** | **T3** | **N2** | **IIIA** | **Proximal** | **8.5** | **well** | **25** | **30** |
| **98** | **75** | **female** | **T4a** | **N3b** | **IIIC** | **Body** | **5.5** | **poor** | **10** | **14** |
| **99** | **57** | **male** | **T4b** | **N3a** | **IIIC** | **Antrum** | **7.8** | **poor** | **15** | **20** |
| **100** | **56** | **female** | **T4b** | **N3b** | **IIIC** | **Antrum** | **4** | **poor** | **11** | **13** |
| **101** | **64** | **male** | **T3** | **N1** | **IIB** | **Proximal** | **9.4** | **poor** | **30** | **37** |
| **102** | **48** | **male** | **T3** | **N3a** | **IIIB** | **Proximal** | **13** | **moderate** | **33** | **39** |
| **103** | **61** | **female** | **T4a** | **N2** | **IIIA** | **Multiple** | **3.2** | **poor** | **no** | **survival** |
| **104** | **54** | **female** | **T4b** | **N2** | **IIIB** | **Antrum** | **1.1** | **poor** | **16** | **19** |
| **105** | **57** | **male** | **T4a** | **N1** | **IIIA** | **Proximal** | **11** | **poor** | **28** | **36** |
| **106** | **53** | **female** | **T4b** | **N3b** | **IIIC** | **Antrum** | **9** | **poor** | **no** | **survival** |
