# Supplementary material for: HOTTIP Predicts Poor Survival in Gastric Cancer Patients and Contributes to Cisplatin Resistance by Sponging miR-216a-5p
Source: Front Cell Dev Biol. 2020 May 8;8:348. doi: 10.3389/fcell.2020.00348 (PMC7225723; doi:10.3389/fcell.2020.00348)
Supplement: TABLE S2 — The sequences of RT-qPCR primer. [file Table_2.docx]

**Table S2** The sequences of RT-qPCR primer

| Prime name | 5’-3’ sequence |
| --- | --- |
| HOTTIP-F | 5’-CCTAAAGCCACGCTTCTTTG-3’ |
| HOTTIP-R | 5’-TGCAGGCTGGAGATCCTACT-3’ |
| GAPDH-F | 5’-TGCACCACCAACTGCTTAGC-3’ |
| GAPDH-R | 5’-GGCATGGACTGTGGTCATGAG-3’ |
| UBC-F | 5’- GATTTGGGTCGCGGTTCTT-3’ |
| UBC-R | 5’- TGCCTTGACATTCTCGATGGT-3’ |
| Bcl-2-F | 5’-GAACTGGGGGAGGATTGTGG-3’ |
| Bcl-2-R | 5’-CCGTACAGTTCCACAAAGGC-3’ |
