# Supplementary material for: HOTTIP Predicts Poor Survival in Gastric Cancer Patients and Contributes to Cisplatin Resistance by Sponging miR-216a-5p
Source: Front Cell Dev Biol. 2020 May 8;8:348. doi: 10.3389/fcell.2020.00348 (PMC7225723; doi:10.3389/fcell.2020.00348)
Supplement: TABLE S3 — The sequences of the siRNA primers. [file Table_3.docx]

**Table S3** The sequences of the siRNA primers

(1) Si-HOTTIP sequences (lnc3180228083948)

| si-HOTTIP-1 | 5’-GCTGCTTTAGAGCCACATA-3’ |
| --- | --- |
| si-HOTTIP-2 | 5’-CCAGCTGCGAATTCTTAAT-3’ |
| si-HOTTIP-3 | 5’-CCTTGATATGCACGCATAT-3’ |
| ASO-HOTTIP -1 | 5’- TCCCAGATAGCATCACATCA-3’ |
| ASO-HOTTIP -2 | 5’-TGCCAGGTTCGCATGTCCTC-3’ |
| ASO-HOTTIP -3 | 5’-CCTCAGTCCTTAGTGTGTCC-3’ |

(2) ATG5 inhibitor sequences

| ATG5 siRNA | GUCCAUCUAAGGAUGCAAUTT |
| --- | --- |

(3) miR-216a-5p inhibitor sequences

| miR-216a-5p inhibitor | UAA UCU CAG CUG GCA ACU GUG A |
| --- | --- |
