# Supplementary figures and images for: HOTTIP Predicts Poor Survival in Gastric Cancer Patients and Contributes to Cisplatin Resistance by Sponging miR-216a-5p
Source: Front Cell Dev Biol. 2020 May 8;8:348. doi: 10.3389/fcell.2020.00348 (PMC7225723; doi:10.3389/fcell.2020.00348)

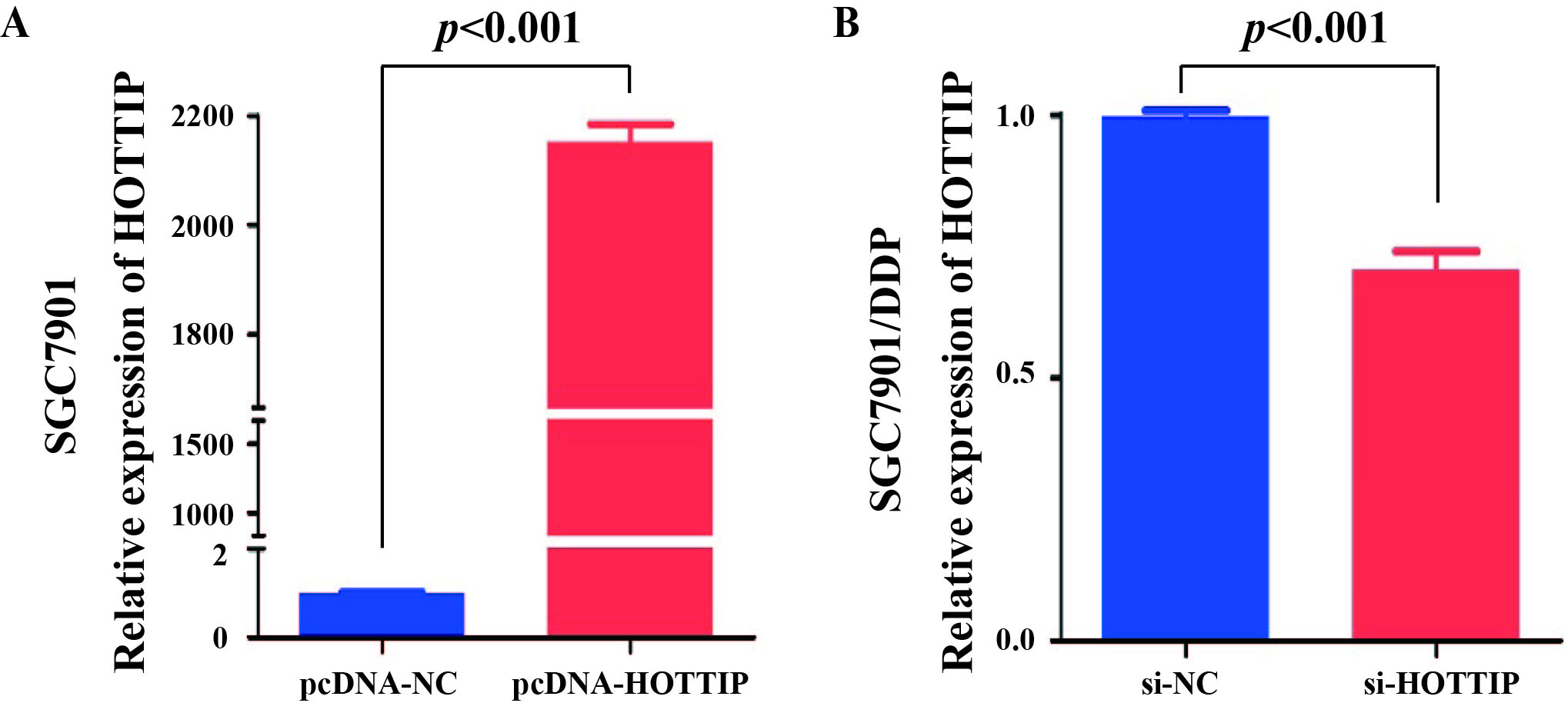

Supplement: FIGURE S1 — HOTTIP overexpression or silencing efficiency. (A) HOTTIP overexpression efficiency in SGC7901 cells. (B) HOTTIP silencing efficiency in SGC7901/DDP cells. [file Image_1.JPEG]

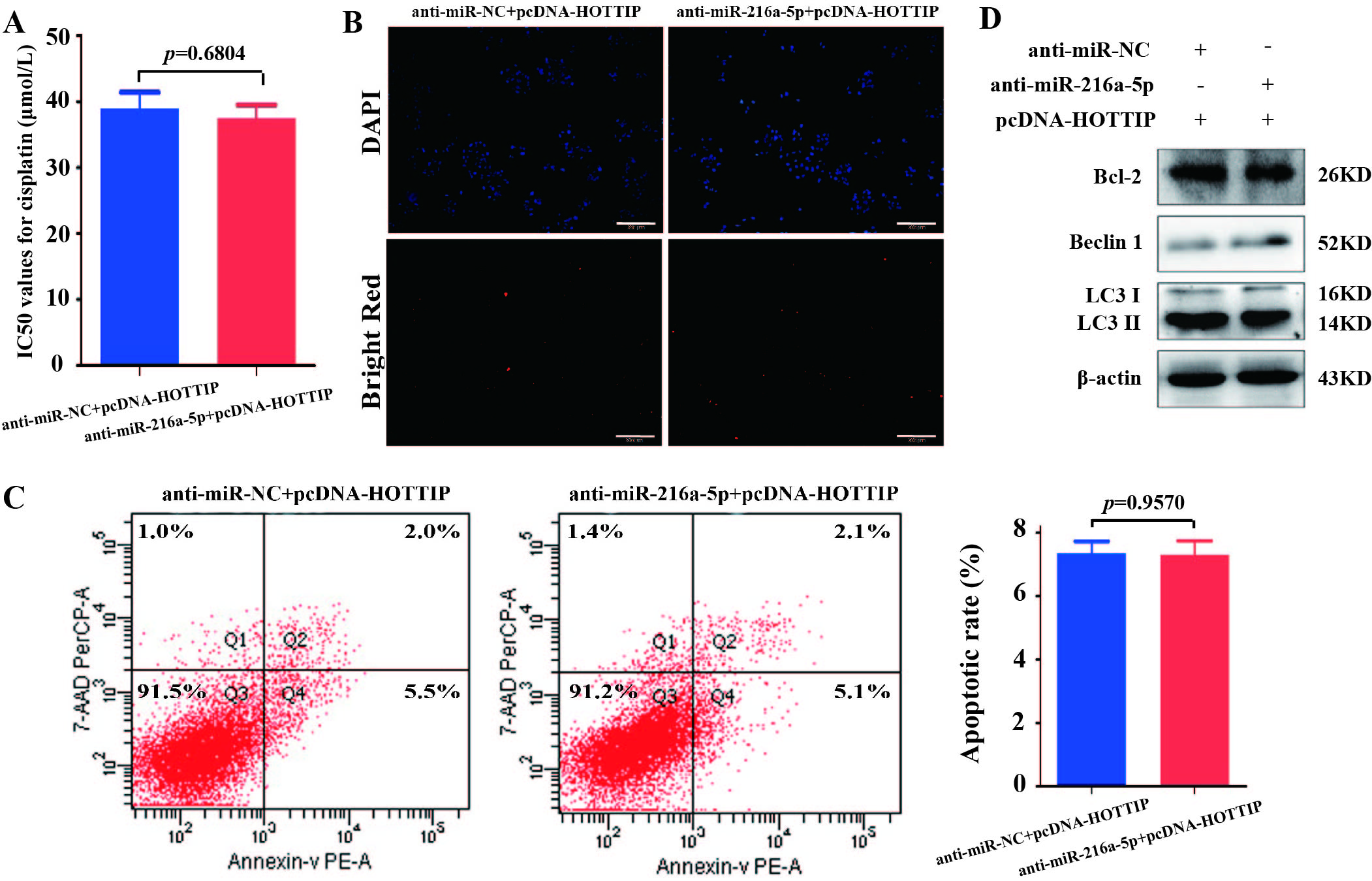

Supplement: FIGURE S2 — HOTTIP directly interacts with miR-216a-5p to play key roles in gastric cancer. (A) The IC50 values for cisplatin of SGC7901 following transfection of anti-miR-NC + pcDNA-HOTTIP or anti-miR-216a-5p + pcDNA-HOTTIP, respectively (IC50 = 38.92 μmol/l vs. 37.50 μmol/l, respectively, p = 0.6804). (B,C) TUNEL (B) and flow cytometry (C) apotosis analysis showed the apoptosis ability in SGC7901 following transfection of anti-miR-NC + pcDNA-HOTTIP or anti-miR-216a-5p + pcDNA-HOTTIP, respectively. Scale bar for TUNEL staining: 200 μm. (D) The relative expression of Bcl-2 protein in SGC7901 following transfection of anti-miR-NC + pcDNA-HOTTIP or anti-miR-216a-5p + pcDNA-HOTTIP, respectively. [file Image_2.JPEG]
